# Remembering Craig Murdoch Pratt, MD, FACC: July 18, 1945 – August 28, 2021

**DOI:** 10.14797/mdcvj.1063

**Published:** 2021-12-15

**Authors:** Shaun Smithson

**Affiliations:** 1 Miami-Dade Cardiology Consultants, Miami, Florida, US

**Keywords:** mentor, cardiologist, leader

## Abstract

Long-time Houston Methodist cardiologist and *Methodist DeBakey Cardiovascular Journal* editorial board member Craig Pratt, MD, FACC, passed away this summer after a long battle with Parkinson’s disease. He is preceded in death by his parents, John and Ruth Neilsen Pratt, and his brother, John Pratt. He is survived by his beloved wife, Carmen; son, Alberto, and his wife Michelle; daughter, Amanda, and her husband Corey; son, Nicolas; and four granddaughters, Catalina, Carmen, Mila, and Charlotte.

In his five-decade career, Pratt was a leader in clinical and research cardiology, serving as director of the Houston Methodist coronary intensive care unit (1978-2011), ambulatory ECG monitoring & stress testing laboratories (1978-2020), and ECG laboratory (2003-2020), and director of research and program director of cardiovascular diseases at Houston Methodist DeBakey Heart & Vascular Center. A keen researcher, Pratt focused on the analysis and treatment of ventricular and supraventricular arrhythmias.

However, one of his most lasting achievements is as an educator. For many years, he supervised the educational programs in the Houston Methodist coronary care unit, training hundreds of residents and fellows in clinical core curricula and encouraging them to participate in clinical trials. For close to four decades, Dr. Pratt ran a weekly clinical research/journal review conference where he taught fellows how to design clinical trials and critically review published clinical research publications. He served as vice chairman of education and program director for the cardiology department for more than a decade. His students and teaching colleagues recognized him with over a dozen faculty and teaching awards for excellence in and commitment to education. One of those students, Dr. Shaun Smithson, delivered a touching eulogy at Dr. Pratt’s funeral. The editors of this journal are honored to print Dr. Smithson’s tribute to his—and our—mentor, colleague, and friend.

## IN MEMORY OF DR. CRAIG PRATT

Good morning, ladies and gentlemen. Today we gather in the midst of a pandemic to honor the life and memory of Dr. Craig Murdoch Pratt.

**Figure:**
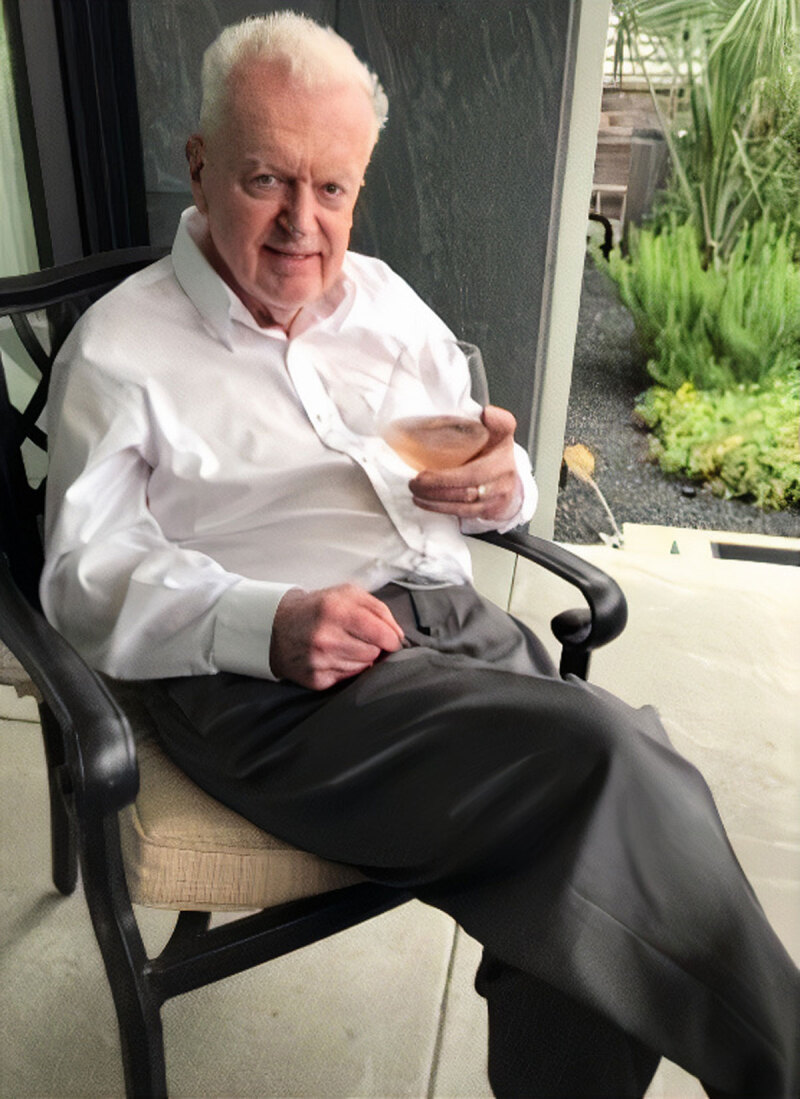
Craig Murdoch Pratt, MD, FACC. Image courtesy of the Pratt family.

Words cannot encompass Dr. Craig Pratt’s legacy, his contribution to medicine (specifically the field of cardiology), his dedication to teaching, nor my relationship with him as a mentor and friend. Similarly, his impressive biography could never capture the true essence of the man.

What I loved most about Dr. Pratt was his humility. Although far removed from his home state, he always had his “California cool.” Dr. Pratt would simply say, “Call me Craig.”

Like many people he influenced, I first met Craig during my cardiac care unit (CCU) rotation as an intern at Houston Methodist Hospital. I remember being so nervous that I always froze up when he called on me. However, right away I sensed that his questions were not a brass demonstration of his own knowledge but an avenue to bring out the best in me and everyone he taught. That’s exactly what teachers do: They find the best way for you to enhance your knowledge. His way was decisive intellect backed by rigorous clinical trial data and infused with his satiric sense of humor.

Craig’s passions were teaching and research. His manuscript on how to interpret a clinical trial is a staple for anyone passing through the CCU at Methodist and should be a requirement before embarking in any research endeavor. The document, like Craig, is timeless. I continue to use it today to teach my own trainees.

When I became a fellow, my relationship with Craig evolved not just because of training but our mutual admiration of Craig’s other passion, sports. He was routine oriented and always had the *Wall Street Journal* and the *Houston Chronicle* open to the sports pages while we drank espresso in the mornings. I can hear him now condemning the Texans, Astros, and the Rockets because of their perennial misfortunes. I smile when I remember him walking around with his iced tea and research documents to review. I remember when I was chief fellow trying to bring urgent matters to his attention and he would say, “That’s great…but when is Usain Bolt running again?”

Craig reminded me that there is more to life than medicine. He educated me about investing, relationships, and cuisines. The first time I ever left the hospital during the day was to eat lunch with him at one of his favorite French restaurants, Café Rabelais in Rice Village. He taught me not only cardiology but about life, and for that I will always be grateful. Craig was the true definition of a mentor. Mentors not only teach but also actualize; they make you reach a potential that you didn’t even think you possessed. When we were both addressed a question, he would always say, “Ask my chief fellow.”

They say a rising tide lifts all boats. Craig was that tide, lifting those of us around him; he reveled in watching people succeed. I’m sure many people in this room would not be in the professional position they are in without Craig. Even after I left Methodist, Craig always ensured I was doing well. Our phone conversations about sports, my job, and checking on his past trainees are moments I will cherish forever.

I feel immensely fortunate to have developed a true friendship with Craig and privileged to spend some personal time with him after he retired, while getting to know his lovely wife Carmen and his family.

As cardiologists, we are trained to work on hearts and know the intricate pathophysiology of complex cardiac disease. Yet the journey to acquire such knowledge about this vital organ can make us seem cold and impersonal. Craig was unique in that he had the knowledge and heart to pass it on with true love and professionalism while keeping his “California cool.” Craig was the true embodiment of a Methodist cardiologist: a leader, teacher, researcher, and clinician, all while demonstrating immense humility. I truly hope that, in this lifetime, I’m able to make a fraction of the contribution to the many lives that he touched. Craig, as always, I will continue to work to make you proud. We miss you dearly.

**Figure:**
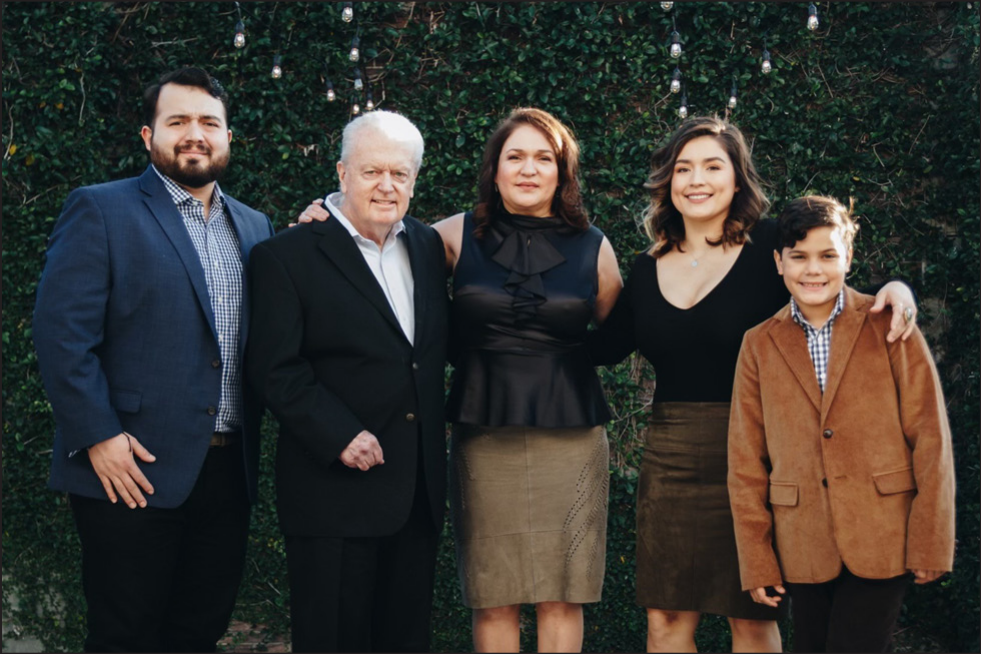
Dr. Craig Pratt with his family. Image courtesy of the Pratt family.

